# Editorial: Tales from across the psychosis spectrum: understanding differences and similarities in mechanisms and experiences

**DOI:** 10.3389/fpsyt.2024.1513000

**Published:** 2024-11-20

**Authors:** Emma Claire Palmer-Cooper, Lyn Ellett, David Benrimoh

**Affiliations:** ^1^ School of Psychology, Southampton Psychosis and Bipolar Research and Innovation Group, Centre for Innovation in Mental Health, University of Southampton, Southampton, United Kingdom; ^2^ Douglas Research Center, Montreal, QC, Canada; ^3^ Department of Psychiatry, McGill University, Montreal, QC, Canada

**Keywords:** psychosis, hallucinations, delusions, biological factors, psychological factors, social determinants, prodromal psychosis

Psychotic experiences exist on a continuum, varying in severity, frequency, and functional disruption (1), across multiple conditions, both as primary and co-morbid diagnoses. These experiences can also manifest in the general population, for example as non-distressing hallucinations or brief occurrences of paranoid thoughts, giving rise to the concept of a psychosis spectrum. At one end sit the experiences many people have - for example, hearing one’s name being called when no one has called it, or believing that other people are trying to annoy or upset you - with the severity and distress caused by these experiences increasing as one moves towards the clinical end of the spectrum. The spectrum is underpinned by a body of research addressing the many factors influencing psychosis, which attempts to understand how this rich tapestry of experience is woven together. In this special edition, several authors have made important contributions to this literature.

In their study evaluating the prevalence of psychosis within a national sample in the United States. Sankoh et al. highlight the complex socio-cultural relationship between psychosis prevalence and ethnic or racial background. Individuals self-identifying as Black and Hispanic were nearly twice as likely to experience psychosis as individuals from White ethnic backgrounds, but had lower rates of comorbid mental illness. Surprisingly, individuals from Black and Hispanic backgrounds had lower overall rates of mental illness. The authors suggest this paradoxical finding may be related to underreporting of other mental illness experiences in ethnically minoritised groups due to stigma, which may lead to delays or avoidance of seeking help. This could explain the greater likelihood of individuals from Black and Hispanic backgrounds experiencing serious mental health problems like psychosis before seeking help, compared to individuals from White backgrounds, who may seek help earlier or more often. This study is an important reminder that measuring phenomenology across the psychosis spectrum intersects with the social determinants of health in important ways.

Focussing on the combined impact of perceptions and experiences of psychosis, O’Brien-Venus et al. investigated how people who hear distressing voices feel dehumanised. Dehumanisation was experienced on a continuum, with personal, social and environmental factors influencing the degree to which individuals felt the loss or reclamation of feeling human. Factors influencing the degree to which individuals felt human included sense of self-worth, agency, belonging, trust in the self, and subjective experience of hearing voices as distressing or harmless. Additionally, feelings of dehumanisation (rather than subjective experience of hearing voices) were more strongly identified as occurring at the ‘end of the continua’ by participants. Participants reported a ‘push and pull’ of these influences moving them up or down the spectrum in response to internal experiences such as the content of voices they heard, and interpersonal responses to these (for example, social rejection and stigma versus acceptance).

Hansson et al. highlighted the critical role of connection and family involvement in psychosis treatment. Interviews highlighted that people with psychosis found systematic family involvement in treatment led to increased knowledge about psychosis through psychoeducation for both individuals with psychosis and family members. This was accompanied by improved understanding of one another’s perspectives and experiences, which led to better interpersonal interactions. This in turn led to better perceived support for the person with psychosis and for the family members supporting them. Having a dedicated space, with structure and boundaries within which to explore information, along with thoughts and feelings of individuals with psychosis and their families were noted as a positive. However, patient hesitancy toward family involvement and a lack of tailored approaches were noted as areas for improvement, along with earlier referral to this intervention. Echoing Hansson et al.’s findings, in previous work, we have argued that specialty care teams in psychosis may operate in part by helping patients better understand and make us of information in the world around them- including improving communication with family (2).

Finally, Amir et al. investigated the complex interaction between biopsychosocial factors and psychosis, comparing clinical high-risk (CHR-P) individuals to those with genetic risk (22q11.2 deletion syndrome). Results demonstrated that CHR-P individuals experienced increased positive psychosis symptoms, dysphoric mood, social functioning, social anhedonia, and a higher IQ than individuals at increased genetic risk. Findings also highlighted that genetic versus clinical risk had a differential impact on substance misuse. CHR-P participants were more likely to use tobacco, alcohol, and cannabis compared to controls. Conversely, individuals at increased genetic risk were less likely to use these substances than controls, which was linked to neurobehavioral factors associated with to 22q11.2 deletion (including lower global social functioning and increased incidence of autism spectrum disorders). This study emphasises that the profiles of those at risk for psychosis can differ greatly, suggesting that the spectrum is not a singular left-right trajectory, but rather a manifold of trajectories and potential subgroups which have yet to be elucidated.

Overall, the articles in this Research Topic highlight complexities that need to be addressed in the field of psychosis research, and especially early and prodromal psychosis. They highlight the importance of understanding how biopsychosocial influences interact in the onset, help-seeking, diagnosis, and treatment of psychosis. These factors need to be carefully considered when designing research protocols and sampling strategies as they may deeply impact the representativeness of the samples collected and, unaddressed, may lead to biased or inaccurate conclusions. More research is needed to understand how social and biological influences interact, how this interaction changes along the continuum, and where on the continuum intervention is likely to be most impactful. Ultimately, larger, more densely temporally sampled studies of psychosis development, sampling from across the continuum and employing a combination of traditional (e.g. questionnaire, imaging, interview) as well as novel computational measures aimed at parsing underlying differences in information processing (3) between potential subgroups on the continuum, may be necessary to fully capture the complexity of the psychosis continuum (see Benrimoh et al. (4) for discussion).
